# ﻿Two new species of *Meliola* from Yunnan Province China

**DOI:** 10.3897/mycokeys.121.158055

**Published:** 2025-08-21

**Authors:** Muhammad Binyamin Khan, Xiang-Yu Zeng, Entaj Tarafder, Sardar Ali, DE-Ping Wei, Ting Chi Wen

**Affiliations:** 1 State Key Laboratory of Green Pesticide, Key Laboratory of Green Pesticide and Agricultural Bioengineering, Ministry of Education, Guizhou University, Guiyang 550025, China Guizhou University Guiyang China; 2 Engineering Research Center of Southwest Bio-Pharmaceutical Resources, Ministry of Education, Guizhou University, Guiyang 550025, China Guizhou University Guiyang China; 3 School of Pharmacy, Guizhou University, Guiyang 550025, China Guizhou University Guiyang China; 4 Department of Plant Pathology, College of Agriculture, Guizhou University, Guiyang, China Guizhou University Guiyang China

**Keywords:** Black mildews, fungal diversity, new species, phylogenetic analysis, taxonomy

## Abstract

*Meliola* is an epifoliar fungal genus commonly known as black mildews, predominantly found in tropical and subtropical regions and considered host specific. This study describes two novel species, *Meliola fuscobrunnea* and *M. fusconigra*, collected from Yunnan Province, China. *Meliola fuscobrunnea* was collected from the leaves of *Annona squamosa*, while *M. fusconigra* was collected from *Xylopia aethiopica*. Comprehensive morpho-molecular analyses were conducted to distinguish these taxa from other *Meliola* species. Phylogenetic analyses based on small subunit (SSU) and large subunit (LSU) rDNA sequences confirmed their placements within *Meliola* and supported their status as distinct lineages. The morpho-molecular data provide robust evidence to establish these species as novel taxa, enriching the current understanding of fungal diversity in Yunnan Province.

## ﻿Introduction

*Meliola* was introduced by Fries (1825), and later, Martin (1941) established Meliolaceae based on *Meliola* as the generic type (Hawksworth and Eriksson 1986; Hongsanan et al. 2015; Hyde et al. 2024). Lumbsch and Huhndorf (2010) placed Meliolales in Sordariomycetes. Kirk et al. (2008) reported over 1,200 species of *Meliola*, and the genus presently contains 2,365 species and 701 varieties (Index Fungorum 2025, accessed April 2025). *Meliola* is one of the most important genera and usually occurs on leaves, petioles, twigs, and fruits (Hosagoudar 2003; Zeng et al. 2017; Jayawardena et al. 2020; Santos et al. 2021; Bermúdez Cova et al. 2022; Zeng et al. 2022; Khan et al. 2025). Despite its morpho-species richness, *Meliola* faces fundamental classification challenges, primarily due to the traditional reliance on host specificity for species delimitation (Zeng et al. 2019, 2020). This practice has led to the description of numerous species based solely on host association, often without adequate morphological or molecular evidence (Justavino and Piepenbring 2007; Marasinghe et al. 2020).

However, host specificity cannot be accepted as a reliable taxonomic criterion, as certain *Meliola* species may occur on multiple hosts, while morphologically similar but genetically distinct species may co-occur on the same host (Santos et al. 2021; Khan et al. 2025). Morphologically, *Meliola* species are characterized by superficial black colonies; globose to subglobose ascomata with superficial mycelia; 2–4-spored, unitunicate asci; and 3–4-septate brown ascospores (Pinho et al. 2012a; Hongsanan et al. 2015; Justavino et al. 2015; Hyde et al. 2020a, 2020b). According to phylogenetic analyses based on LSU and ITS sequences, the placement of *Meliola* has been confirmed in Sordariomycetes, although it appears to be polyphyletic due to limited molecular data (Gregory and John 1999; Pinho et al. 2012a, 2012b; Hongsanan et al. 2015; Justavino et al. 2015; Hyde et al. 2020b; Marasinghe et al. 2020; Zeng et al. 2020). The species of *Meliola* are usually biotrophic and difficult to culture using artificial media. Therefore, direct DNA extraction from fruiting structures is often susceptible to contamination by other fungal species. These persistent issues underscore the urgent need for a comprehensive taxonomic revision of *Meliola*, incorporating detailed morphological examination and multilocus molecular phylogenetic analyses (Hongsanan et al. 2015; Zeng et al. 2017).

This study introduces *Meliola fuscobrunnea* and *M. fusconigra* as new species collected from *Annona squamosa* and *Xylopia aethiopica*, respectively, in Yunnan Province, China. *Xylopia aethiopica* (Dunal) A. Rich. has previously been recorded as a host for *Meliola* species (*M. xylopiae* F. Stevens and *M. kuprensis* Deighton) (Deighton 1951; Hansford 1961). In contrast, *Annona squamosa* L. is reported here for the first time as a host for the genus *Meliola*. Comprehensive morpho-molecular analyses supported their distinct placements within *Meliola*. These findings contribute to the taxonomy of *Meliola*, improve understanding of host-specific plant–fungus interactions, and enrich fungal biodiversity records in an underexplored region of Yunnan Province, China. Moreover, the results provide essential baseline data for future ecological, biogeographical, and evolutionary studies and support global initiatives aimed at documenting fungal diversity in the context of environmental changes.

## ﻿Materials and methods

### ﻿Study area

A field survey was conducted in July 2024 in Yunnan Province, China, a global biodiversity hotspot known for its rich fungal diversity, accounting for over 40% of all fungal species recorded in China (Feng and Yang 2018; Liu et al. 2018; Phookamsak et al. 2024). The study focused specifically on the forests of Yunnan Province, China. This region receives an annual rainfall of 1000–4000 mm and has a distinct seasonal climate, with cold and dry conditions from November to April and warm, humid conditions from May to October (Dumbacher et al. 2011).

### ﻿Morphological characterization

For the morphological observations, infected plant leaves were first examined using a stereomicroscope (LABOMED America, 7GA9, USA). A small amount of fungal material was gently scraped from the leaf surface using a fine needle and mounted in a drop of 5% aqueous KOH on a clean glass slide for observation using a compound microscope (Senanayake et al. 2020). Microscopic features of the mycelium, conidiophores, conidia, and other hyphal characters were examined at magnifications of 4×, 10×, and 40×. Measurements were made with an ocular micrometer at 40×. Light micrographs of microscopic features of the fungal pathogen were also captured.

### ﻿DNA extraction, amplification, and sequencing

Genomic DNA was directly extracted from the isolated fruiting structures of *M. fuscobrunnea* and *M. fusconigra* scraped from the infected leaf surfaces of *A. squamosa* and *X. aethiopica*, respectively, using the E.Z.N.A. fungal DNA kit (Omega Bio-Tek, Norcross, GA, USA). The procedures followed the manufacturer’s protocol, and the extracted DNA was stored at –20 °C. Partial sequences from the SSU region of the nrDNA were amplified under standard PCR conditions using the primer pair NS1/NS4 (White et al. 1990). The LSU region was amplified using the primer pair LR0R/LR5 (Vilgalys and Hester 1990). A 25 μL reaction mixture containing 1.6 μL dNTP mix (2.5 mM/mL), 0.2 μL Taq polymerase (5 U/mL), 2 μL polymerase buffer (10×), 1 μL each of forward and reverse primers (10 mM/mL), and 1 μL DNA template was used for the PCR experiments. Amplifications were carried out in a T100™ Thermal Cycler (BIO-RAD) (Table 1). Sangon Biotech (Shanghai) Co., Ltd. sequenced the PCR products using the same primers used in the amplification reactions.

**Table 1.** The PCR conditions and the primers used in this study.

Locus	Primer	Sequence	PCR condition	References
SSU	NS1	GTAGTCATATGCTTGTTC	(1) Initial denaturation for 3 min at 94 °CC	White et al. (1990)
	NS4	CTTCCGTCAACCTTTAAG	(2) 40 cycles of denaturation at 94 °C for 45 s, annealing at 56 °C for 50 s, and extension at 72 °C for 1 min	
LSU	LROR	ACCCGCTGAACTTTAAGC	(3) Final elongation at 72 °C for 10 min	Vilgalys and Hester (1990)
	LR5	TCCTGAGGGAAACTTCG	(4) Storage at 4 °C	

### ﻿Phylogenetic analysis

**Figure 1.**
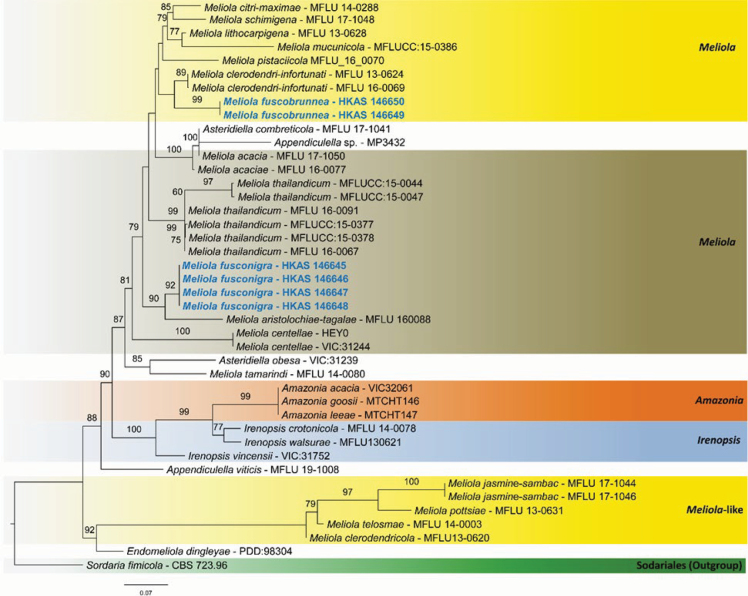
Molecular phylogenetic analysis of *Meliola* species by the maximum likelihood (ML) method based on combined SSU and LSU sequences. The tree is rooted with *Sordaria fimicola* (CBS 723.96). Newly generated type sequences are shown in blue.

The newly generated forward and reverse sequences were assembled using BioEdit v. 7.2.5 (Hall 1999) to generate a consensus sequence. A BLAST search (https://blast.ncbi.nlm.nih.gov) was conducted through the NCBI GenBank database to identify and retrieve reference sequences homologous to *Meliola* sp. The reference sequences were obtained from the literature and GenBank. The sequences (SSU and LSU) were independently aligned using the online MUSCLE version (Edgar 2004), and BioEdit was used for manual editing and refinement. *Sordaria fimicola* (Roberge ex Desm.) Ces. & De Not. (CBS 723.96) was selected as the outgroup taxon (Zeng et al. 2022). Maximum likelihood (ML) phylogenetic analyses were performed using RAxML (Miller et al. 2010) with the GTRGAMMA model, and 1,000 bootstrap replicates were used to assess branch support. Phylogenetic analyses were conducted on the CIPRES Science Gateway platform, and the resulting phylogenetic tree is presented in Fig. 1. New species are established based on the recommendations outlined by Jeewon and Hyde (2016).

## ﻿Results

### ﻿Phylogenetic analyses

The newly generated sequences were deposited in GenBank, and their accession numbers are provided in Table 2. The combined dataset for phylogenetic analysis incorporated nuclear ribosomal loci (SSU and LSU) representing 42 strains, with *Sordaria fimicola* CBS 723.96 (Sordariaceae, Sordariales) designated as the outgroup taxon. The RAxML analysis of the combined dataset yielded the best scoring tree (Fig. 1), with a final ML optimization likelihood value of –18855.431359.

**Table 2.** Fungal species used for phylogenetic analyses of *Meliola*. Newly generated sequences from this study are shown in bold, while “–” indicates data unavailable.

Species	Strain	Genbank accession number		Country	References
		SSU	LSU		
*Amazonia acaciae*	VIC32061	KC618656	-	Brazil	Pinho et al. 2012b
*Amazonia goosii*	MTCHT146	OM296101	-	India	Hosagoudar and Abraham (1998)
*Amazonia leeae*	MTCHT147	OM296099	-	India	Hansford and Thirumalachar (1948)
*Appendiculella* sp.	MP3432	DQ508301	-	Panama	Rodríguez and Piepenbring (2007)
*Appendiculella viticis*	MFLU 19-1008	-	MT108888	Thailand	Marasingheet al. 2020
*Asteridiella combreticola*	MFLU 17-1041	MN747498	-	Thailand	Zeng et al. 2022
*Asteridiella obesa*	VIC 31239	KC618653	NG057014	Brazil	Pinho et al. 2012b
*Endomeliola dingleyae*	PDD:98304	-	GU138866	New Zealand	Hughes and Pirozynski (1994)
*Irenopsis crotonicola*	MFLU:14-0078	KY554796	-	Thailand	Zeng et al. 2018
*Irenopsis vincensii*	VIC:31752	-	JX133163	Brazil	Zeng et al. 2018
*Irenopsis walsurae*	MFLU13-0621	MN747487	-	Thailand	Zeng et al. 2018
Meliola fuscobrunnea	HKAS 146650	PV299283	PV298265	China	This study
Meliola fuscobrunnea	HKAS 146649	PV298266	PV298264	China	This study
Meliola fusconigra	HKAS 146645	PV298261	PV298245	China	This study
Meliola fusconigra	HKAS 146646	PV299282	PV446596	China	This study
Meliola fusconigra	HKAS 146647	PV298262	PV297977	China	This study
Meliola fusconigra	HKAS 146648	PV298263	PV454349	China	This study
*Meliola acacia*	MFLU 16-0077	NG_077426	-	Thailand	Zeng et al. 2020
*Meliola aristolochiae-tagalae*	MFLU 160088	MN747496	-	Thailand	Zeng et al. 2020
*Meliola centellae*	VIC:31244	-	NG042650	Brazil	Pinho et al. 2012b
*Meliola citri-maximae*	MFLU 14-0288	NG_070325	-	Thailand	Hyde et al. 2017
*Meliola clerodendricola*	MFLU13-0620	MN747486	-	India	Minter 2006
*Meliola clerodendri-infortunati*	MFLU:13-0624	NG_070324	-	Thailand	Zeng et al. 2017
*Meliola clerodendri-infortunati*	MFLU 16-0069	-	MN788607	Thailand	Zeng et al. 2017
*Meliola jasmini-sambac*	MFLU 17-1044	MN747499	MT911464	Thailand	Zeng et al. 2020
*Meliola jasmini-sambac*	MFLU 17-1046	MN747500	-	Thailand	Zeng et al. 2020
*Meliola lithocarpigena*	MFLU 13-0628	NG_077423	-	Thailand	Zeng et al. 2020
*Meliola pistaciicola*	MFLU 16-0070	NG_077425	-	Thailand	Zeng et al. 2020
*Meliola pottsiae*	MFLU 13-0631	NG_077424	-	Thailand	Zeng et al. 2020
*Meliola tamarindi*	MFLU:14-0080	KY554797	-	Philippines	Zeng et al. 2017
*Meliola telosmae*	MFLU 14-0003	MK103390	-	Thailand	Zeng et al. 2020
*Meliola thailandicum*	MFLU 16-0067	-	MN788606	Thailand	Hongsanan et al. 2015
*Meliola thailandicum*	MFLUCC:15-0378	-	KR868695	Thailand	Hongsanan et al. 2015
*Meliola thailandicum*	MFLUCC:15-0047	-	KR868698	Thailand	Hongsanan et al. 2015
*Sordaria fimicola*	CBS 723.26	-	MH874231	Papua New Guinea	Zeng et al. 2020

Phylogenetic analyses revealed that our strains were placed within *Meliola* and clustered independently from other species. *Meliola fuscobrunnea* (HKAS 146649, HKAS 146650) forms a strongly supported clade with *M. clerodendri-infortunati* X.Y. Zeng, K.D. Hyde and T.C. Wen (MFLU 13-0624, MFLU 16-0069), with high statistical support (MLBS = 99%), indicating a close phylogenetic affinity between these taxa. *Meliola fusconigra* (HKAS 146645, HKAS 146646, HKAS 146647, and HKAS 146648) formed a well-supported clade (MLBS = 92%), closely related to *Meliola aristolochiae-tagalae* X.Y. Zeng, K.D. Hyde and T.C. Wen (MFLU 160088), with strong support (MLBS = 90%), suggesting a close evolutionary relationship between the two species (Fig. 1).

### ﻿Taxonomy

#### ﻿ Meliola fuscobrunnea

Taxon classificationFungiMeliolalesMeliolaceae

Khan M.B. & T. C. Wen sp. nov.

08EB5107-3746-537F-B758-1DF4DDCBA281

MycoBank No: 858334

Fig. 2

##### Etymology.

From Latin, “*fusco*” meaning “dark,” and “*brunnea*” meaning “brown,” *referring*” to the distinct dark brown color of Ascospores.

##### Diagnosis.

Appressoria small and variable in size and slightly thick; ascomata superficial and relatively small; setae moderately long, straight to slightly curved with well-developed, clearly visible phialides; ascospores cylindrical or oblong and comparatively large, and dark brown in color.

##### Holotype.

China • Yunnan Province, Gaoligong Mountains, Muhammad Binyamin Khan, 27 July 2024, HKAS 146650, GenBank accession numbers PV299283 (SSU) and PV298265 (LSU).

##### Additional materials examined.

China • Yunnan Province, Gejia Mountain, Muhammad Binyamin Khan, 26 July 2024, HKAS 146649, GenBank accession numbers PV298266 (SSU) and PV298264 (LSU).

##### Habitat.

On leaving leaves of *Annona squamosa*.

##### Description.

Biotrophic on the surface of living leaves of *A. squamosa*. ***Colonies*** 5–10 mm in diameter, epiphyllous, scattered, black. ***Hyphae*** superﬁcial, black, straight to substraight, branched, septate, each cell 19–21 μm long (x̄ = 20 μm, n = 20), reticulate with dark brown setae. ***Hyphal setae*** up to 305 μm long, subdense, dark brown, straight to curved, 2-dentate, obtuse. ***Appressoria*** 8–18 × 4–11 μm (x̄= 13 × 7 µm, n = 30), 2-celled, brown, clavate, straight to curved, formed near the septa, unilateral, antrorse. Sexual morph: ***Ascomata*** up to 200 μm in diameter, superﬁcial, subdense, dark, globose to subglobose, with a central ostiole. ***Peridium*** comprises hyaline inner cell and dark brown outer wall with textura angularis. ***Hamathecium*** with evanescent paraphyses. ***Ascospores*** 46–52 × 14–18 μm (x̄= 49 × 16 µm, n = 20), 2–3 seriate, cylindrical or oblong, hyaline when young, becoming dark brown when mature, 4-septate, constricted at the septa, rounded at both ends, smooth-walled. Asexual morph: ***Phialides*** 17–25 × 5–9 μm (x̄= 21 × 77 µm, n = 10), opposite to unilateral, flask-shaped, mixed with appressoria, ampulliform.

##### Notes.

*Meliola fuscobrunnea* (HKAS 146649, HKAS 146650) forms a strongly supported clade with *M. clerodendri-infortunati* (MFLU 13-0624, MFLU 16-0069) with high statistical support (MLBS 99%) (Fig. 1). Both species have similar orientation and arrangement of superficial hyphae, hyphal setae length, appressorium colour and position, ascomata shape, and peridium wall colour. *Meliola clerodendri-infortunati* is distinct from *M. fuscobrunnea* in hyphal setae apex and size of appressorium, ascomata, and ascospore. Additionally, the two species also differ in host association, with the former found on *Clerodendrum infortunatum* L. and the latter on *A. squamosa* (Hyde et al. 2017). Hence, based on the differences in morphological characteristics, phylogenetic analyses, and host differences, we introduce *M. fuscobrunnea* as a new species of *Meliola*.

**Figure 2.**
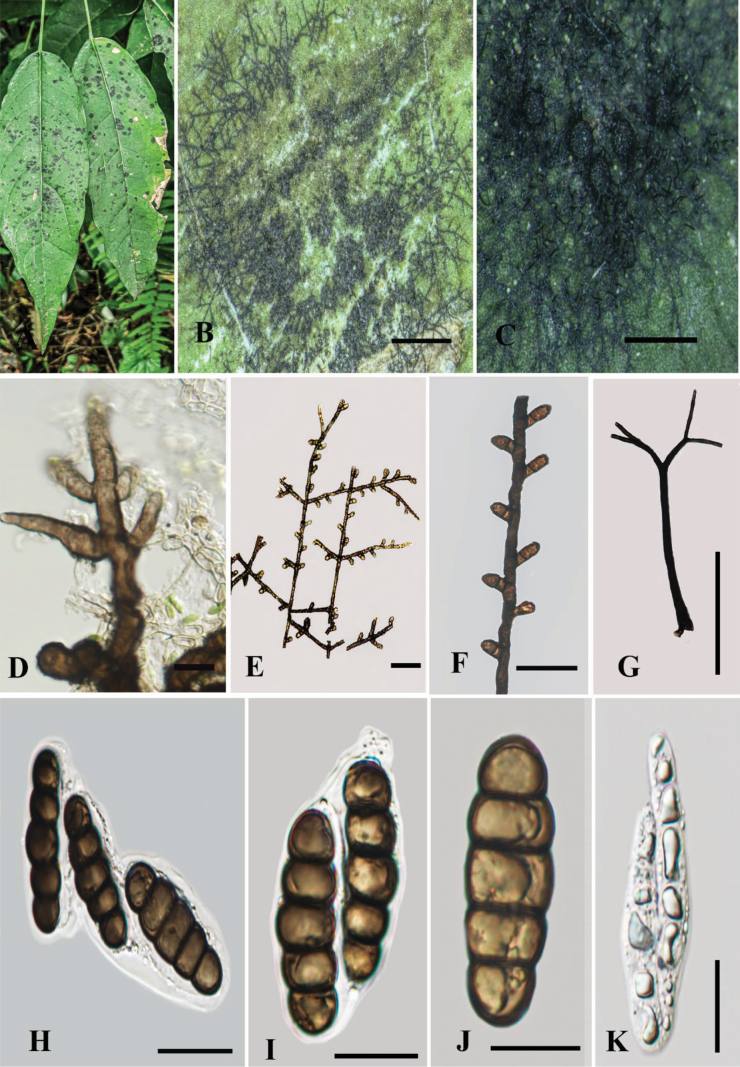
*Meliola fuscobrunnea* A. Host leaves; B. Colonies on the leaf surface; C. Ascomata on leaf surface; D. Phialides; E, F. Reticulate superficial hyphae with appressoria; G. Hyphal setae; H, I. Asci; J. Ascospore; K. Immature asci. Scale bars: 1 mm (B); 10 (D, E); 20 um (F–K).

#### ﻿ Meliola fusconigra

Taxon classificationFungiMeliolalesMeliolaceae

Khan M.B. & T. C. Wen sp. nov.

E062EE16-4EF9-5194-82B1-DC3877E54EC9

MycoBank No: 858335

Fig. 3

##### Etymology.

From Latin, “*fusco*” meaning dark and “*nigra*” meaning “black,” referring to the distinct dark black colonies of species on natural substrate.

##### Diagnosis.

Appressoria clavate, brown, unilateral, and antrorse; ascomata globose to subglobose with a central ostiole. Phialides flask-shaped, well-developed, and mixed with appressoria; ascospores cylindrical to oblong, large, smooth-walled, and dark brown at maturity.

##### Holotype.

China • Yunnan Province, Laifengshan National Forest Park, Muhammad Binyamin Khan, 26 July 2024, HKAS 146645, GenBank accession numbers PV298261 (SSU) and PV298245 (LSU).

##### Additional materials examined.

China • Yunnan Province, Gaoligongshan, Muhammad Binyamin Khan, 27 July 2024, HKAS 146646, HKAS 146647, and HKAS 146648, GenBank accession numbers PV299282, PV298262, and PV298263 (SSU); PV446596, PV297977, and PV454349 (LSU)

##### Habitat.

On leaving leaves of *Xylopia aethiopica*.

##### Description.

Biotrophic on the surface of living leaves of *X. aethiopica*. Colonies 4–8 mm in diameter, epiphyllous, dense to subdense, dark black. ***Hyphae*** superﬁcial, black, straight to substraight, branched, black dark at septa, each cell 18–88 μm long (x̄ = 53 μm, n = 10), loosely reticulate, with dark black setae. ***Hyphal setae*** 291–306 × 2–6 μm (x̄= 298 × 4 μm, n = 10), narrowly cylindrical, rounded to acute at the apex. ***Appressoria*** 24–28 × 11–15 μm (x̄= 26 × 13 µm, n = 20), 2-celled, brown, clavate, substraight, formed near the septa, unilateral, antrorse. ***Ascomata*** up to 250 μm in diameter, superﬁcial, subdense, dark brown, globose to subglobose, with a central ostiole. ***Peridium*** comprises hyaline inner cell and dark brown outer wall with textura angularis. ***Hamathecium*** with evanescent paraphyses. ***Ascospores*** 48–60 × 15–21 μm (x̄ = 54 × 18 µm, n = 20), 3–4 seriate, cylindrical or oblong, hyaline when young, becoming dark brown when mature, 4-septate, constricted at the septa, rounded at both ends, smooth-walled. Asexual morph: ***Phialides*** 40–30 × 5–9 μm (x̄ = 35 × 7 µm, n = 10), opposite to unilateral, sometimes alternate, flask-shaped, few mixed with appressoria, ampulliform.

**Figure 3.**
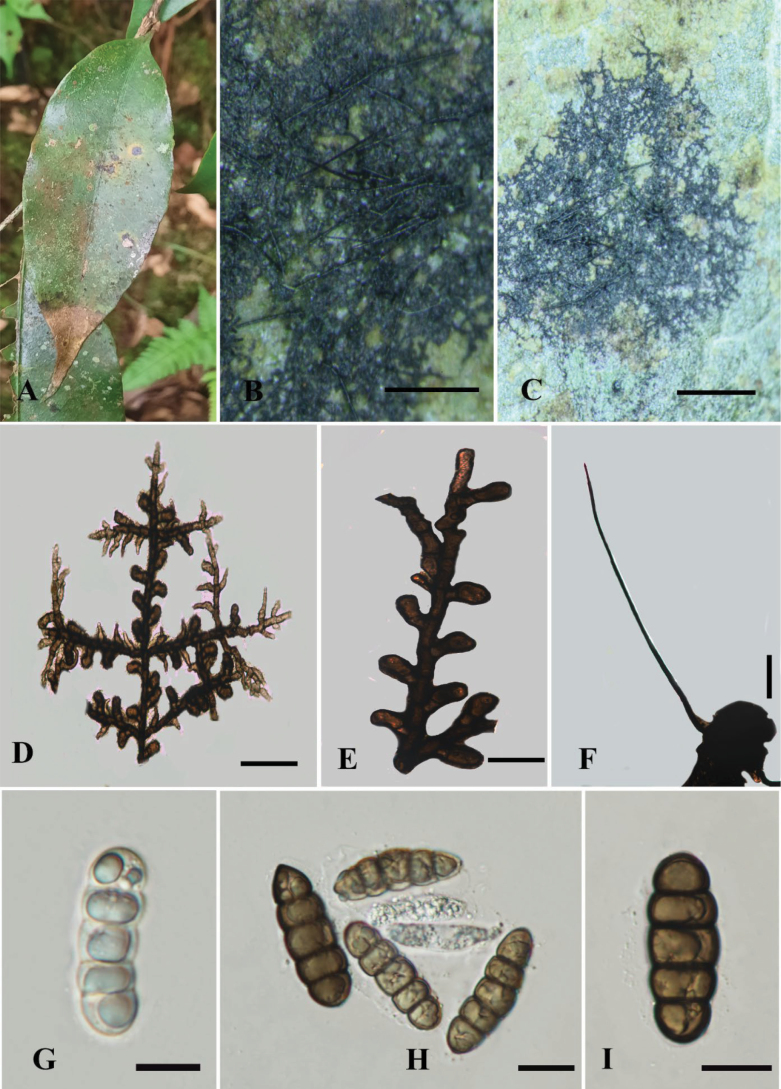
*Meliola fusconigra* A. Host leaves; B, C. Ascomata colony on surface of leaves; D, E. Reticulate hyphae with appressoria; F. Hyphal setae; G–I. Ascospores. Scale bars: 1 mm (B, C); 20 um (D–I).

##### Notes.

Phylogenetic analyses showed that *M. fusconigra* forms a sister clade to *M. aristolochiae-tagalae* with strong statistical support (MLBS = 90%), confirming their close evolutionary relationship (Fig. 1). Both species share similarity in colony and peridium cell colour, orientation of appressoria, and shape of appressoria, ascomata, and ascospore (Zeng et al. 2022). However, notable differences can be observed between *M. fusconigra* and *M. aristolochiae-tagalae* in the colour of superficial hyphae and the size of individual cells of superficial hyphae, appressoria, ascomata, ascospores, and phialides, and host species (Zeng et al. 2022). This morpho-anatomical comparison with the phylogenetically allied species represents *M. fusconigra* as a distinct new species.

## ﻿Discussion

This study proposes two new species, *Meliola fuscobrunnea* and *M. fusconigra*. These species were collected from the mountains of Yunnan Province, China, and were identified as novel taxa based on morpho-molecular evidence. *Annona squamosa* is reported here for the first time as a host for the genus *Meliola*. *Meliola fuscobrunnea* (HKAS 146649, HKAS 146650) forms a strongly supported clade with *M. clerodendri-infortunati* (MFLU 13-0624, MFLU 16-0069) with high statistical support (MLBS = 99%) and differs from *M. clerodendri-infortunati* (MFLU 16-0069) by 70 nucleotide substitutions in the LSU region and by 9 substitutions in the SSU region when compared with the SSU sequence of the same species (NG_070324) (Jeewon and Hyde 2016). Both species have straight to substraight and reticulated superficial hyphae, up to 300 μm long and dark brown hyphal setae, brown and unilateral to antrorse appressorium, globose to subglobose ascomata, and a hyaline inner and dark brown outer wall of peridium. However, the former can be distinguished from the latter by its hyphal setae with acute apex, slightly larger appressoria (14 × 9 µm), slightly smaller ascomata (up to 160 μm diam.), smaller ascospores (39 × 9 µm), and rarity of phialides. Moreover, *M. clerodendri-infortunati* has only cylindrical ascospores, while *M. fuscobrunnea* has oblong ascospores in addition to cylindrical ascospores. *Meliola clerodendri-infortunati* was recovered from the living leaves of *C. infortunatum* (Hyde et al. 2017), while *Meliola fuscobrunnea* was found on the living leaves of *Annona squamosa* in the present study, indicating that the two species might differ in their host association.

*Meliola fusconigra* (HKAS 146645, HKAS 146646, HKAS 146647, and HKAS 146648) formed a well-supported clade (MLBS = 92%), indicating its distinct phylogenetic placement. It is closely related to *Meliola aristolochiae-tagalae* (MFLU 160088), with strong bootstrap support (MLBS = 90%), suggesting a close evolutionary relationship between the two species. It differs from *Meliola aristolochiae-tagalae* (MFLU 160088) by 20 nucleotide substitutions in the SSU region; however, LSU sequence data for *M. aristolochiae-tagalae* were not included in this study (Jeewon and Hyde 2016). *Meliola aristolochiae-tagalae* shares common morphological features with *M. fusconigra*, such as colony color (black), orientation of superficial hyphae (straight to substraight), orientation and shape of appressoria (unilateral and clavate), ascomata shape (globose to subglobose), peridium cell color (hyaline inner cell and dark brown outer wall), and ascospore shape (cylindrical or oblong). However, *M. aristolochiae-tagalae* differs from *M. fusconigra* in having brown, closely reticulated superficial hyphae with relatively smaller individual cells (19 μm long), relatively larger (up to 420 μm), dark brown, and straight hyphal setae, shorter appressoria (17 μm), smaller ascomata (200 μm diam.), smaller ascospores (42 × 14 µm), and 2–3-seriate ascospores, and smaller, unilateral phialides (18 × 8 µm), while *M. fusconigra* contains brown and loosely reticulated superficial hyphae with larger individual cells (53 μm long), smaller (298 μm), black, and narrowly cylindrical hyphal setae, longer appressoria (26 μm), bigger ascomata (250 μm diam.), bigger ascospores (54 × 18 µm), and 3–4-seriate ascospores, and bigger, opposite to unilateral, sometimes alternate phialides (35 × 7 µm). Moreover, both species have different hosts, as the former was reported to infect *Aristolochia tagala* Cham., while the latter was found in association with *X. aethiopica* (Zeng et al. 2022).

This study identified *Meliola fuscobrunnea* and *M. fusconigra* as new species, supported by comprehensive morpho-molecular analyses. Moreover, this study also expands the known host range of *Meliola* by recording *Annona squamosa* as a new host while contributing to the understanding of fungal diversity in Yunnan Province, China.

## Supplementary Material

XML Treatment for Meliola fuscobrunnea

XML Treatment for Meliola fusconigra

## ﻿Additional information

### Use of AI

No use of AI was reported.

### Author ORCIDs

Muhammad Binyamin Khan https://orcid.org/0000-0001-5979-1036

Xiang-Yu Zeng https://orcid.org/0000-0003-1341-1004

Entaj Tarafder https://orcid.org/0000-0002-3680-3433

Sardar Ali https://orcid.org/0009-0006-7938-882X

DE-Ping Wei https://orcid.org/0000-0002-3740-0142

Ting Chi Wen https://orcid.org/0000-0003-1744-5869

### Data availability

All of the data that support the findings of this study are available in the main text.
